# Visual Imagery and Trauma: A PRISMA-Guided Narrative Review on the Neurocognitive and Psychiatric Implications of Aphantasia in PTSD

**DOI:** 10.1192/j.eurpsy.2026.11871

**Published:** 2026-07-31

**Authors:** N. Northover, S. Kolloju, N. Ibrahim, J. Singh, A. Sanchez, S. Prasad, T. Kainth, S. Gunturu

**Affiliations:** 1 Psychiatry, American University of the Caribbean, Cupecoy, Saint Martin (French Part); 2 Psychiatry, Manhattan Psychiatric Center, New York City, United States; 3 Psychiatry, Kasturba Medical College, Mangalore, India; 4 Psychiatry, Nassau University Medical Center, East Meadow; 5 Psychiatry, American University of the Caribbean, Cupecoy; 6 Psychiatry, BronxCare Health System, Bronx, United States

## Abstract

**Introduction:**

Aphantasia, the inability to voluntarily generate mental visual images, may influence emotional processing and memory encoding. While its effects on memory and cognition have been studied, its role in traumatic memory and PTSD remains underexplored. Understanding how aphantasia interacts with PTSD, depression, and social phobia is crucial, underscoring the need for further investigation into this unique neurocognitive phenotype.

**Objectives:**

To examine how absent visual imagery shapes PTSD symptoms, highlight diagnostic challenges, and propose clinical adaptations for imagery differences.

**Methods:**

A narrative review guided by PRISMA methodology was conducted using keyword pairings such as *aphantasia* and *PTSD* across PubMed, Scopus, PsycINFO, and grey literature published between 2010–2025. Of 602 records screened, 44 relevant sources were identified; after deduplication and manual screening, 10 were included (empirical studies, case reports, theoretical reviews).

**Results:**

Distinct PTSD profiles are observed in individuals with aphantasia, as impaired visual imagery limits flashbacks and intrusive memories. In a study of 183 trauma-exposed participants, only 43% of individuals with aphantasia reported flashbacks versus 92.9% of controls, despite similar overall PTSD severity. Instead, they exhibited higher rates of guilt, rumination, and detachment. Another study of 125 participants confirmed lower re-experiencing scores (p < .01) but higher negative mood and cognition (p < .05) in individuals with aphantasia. When imagining fearful scenarios, controls showed autonomic arousal (mean +0.46 μS), whereas individuals with aphantasia showed no significant change (–0.06 μS; p = 0.039). Neuroimaging similarly revealed reduced activation of visual cortices during imagery tasks in aphantasia, reflecting absent sensory re-engagement. Conditioning studies demonstrated reduced fear responses, suggesting that recovery in aphantasia occurs through verbal or conceptual pathways rather than visual reliving.

**Image 1:**
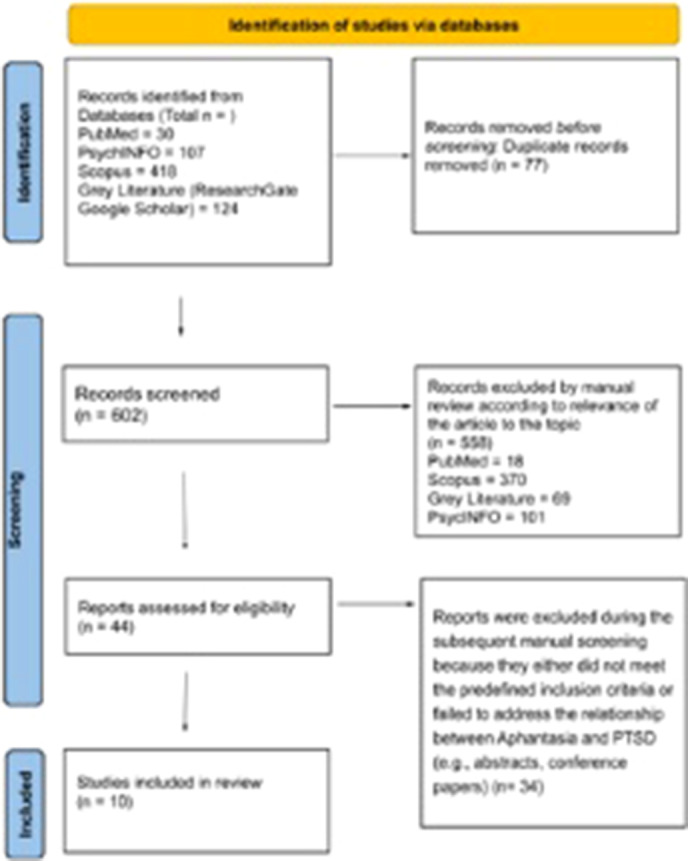
Image 1: Long description.

**Conclusions:**

Aphantasia does not preclude PTSD but may buffer against hallmark symptoms such as flashbacks. Traditional treatments for PTSD, such as prolonged exposure and EMDR, have been reported as less effective in this population highlighting the need to assess mental imagery ability during diagnosis to improve symptom interpretation. Therapies emphasizing cognitive restructuring or somatic experience may be more effective than imagery-based techniques. Further research is warranted to elucidate the neural and clinical mechanisms of aphantasia in PTSD and to integrate imagery capacity into trauma-informed frameworks.

**Disclosure of Interest:**

None Declared

## Long descriptions

### Image 1: Long description

The flowchart is organized into three vertical phases labeled on the left: Identification, Screening, and Included.

1. Identification Phase:

- Top box: Identification of studies via databases.

- Central box: Records identified from Databases (Total n = 679). Breakdown: PubMed = 30, PsycINFO = 107, Scopus = 418, Grey Literature (ResearchGate, Google Scholar) = 124.

- Right box: Records removed before screening: Duplicate records removed (n = 77).

2. Screening Phase:

- Central box: Records screened (n = 602).

- Right box: Records excluded by manual review according to relevance of the article to the topic (n = 558). Breakdown: PubMed = 18, Scopus = 370, Grey Literature = 69, PsycINFO = 101.

- Central box below: Reports assessed for eligibility (n = 44).

- Right box: Reports were excluded during the subsequent manual screening because they either did not meet the predefined inclusion criteria or failed to address the relationship between Aphantasia and P T S D (e.g., abstracts, conference papers) (n = 34).

3. Included Phase:

- Bottom central box: Studies included in review (n = 10).

Navigate back to Image 1:.

